# Vorbeugender Rettungsdienst – präventive Ansätze und Förderung von Gesundheitskompetenz an den Schnittstellen zur Notfallrettung

**DOI:** 10.1007/s00101-023-01272-6

**Published:** 2023-03-13

**Authors:** Florian Breuer, Stefan K. Beckers, Janosch Dahmen, Andre Gnirke, Christopher Pommerenke, Stefan Poloczek

**Affiliations:** 1 Einsatzvorbereitung Rettungsdienst, Berliner Feuerwehr, Berlin, Deutschland; 2 Ärztliche Leitung Rettungsdienst, Berliner Feuerwehr, Berlin, Deutschland; 3 Ärztliche Leitung Rettungsdienst, Rheinisch-Bergischer Kreis, Amt für Feuerschutz und Rettungswesen, Am Rübezahlwald 7, 51469 Bergisch Gladbach, Deutschland; 4 Fakultät für Gesundheit, Department Humanmedizin, Universität Witten/Herdecke, Witten, Deutschland; 5 Ärztliche Leitung Rettungsdienst Stadt Aachen, Fachbereich Feuerwehr und Rettungsdienst Stadt Aachen, Aachen, Deutschland; 6 Ärztliche Leitung Rettungsdienst, Rettungsdienst-Kooperation in Schleswig-Holstein, Pinneberg, Deutschland

**Keywords:** Prähospitale Versorgung, Leitstelle, Notfallmedizin, „Frequent user“, Psychosozialer Notfall, Prehospital care, Emergency medical dispatch center, Emergency medicine, Frequent user, Psychosocial emergency

## Abstract

In den Rettungsdienstgesetzen der Länder beschränken sich die Ausführungen bislang im Wesentlichen auf die Durchführung von Maßnahmen zum Erhalt der Gesundheit von Notfallpatientinnen und Notfallpatienten sowie auf die Beförderung in ein geeignetes Krankenhaus. Der vorbeugende Brandschutz hingegen ist in den Feuerwehrgesetzen bzw. durch Rechtsverordnungen geregelt. Zunehmende Einsatzzahlen im Rettungsdienst und fehlende Einrichtungen der alternativen Versorgung begründen die Notwendigkeit eines vorbeugenden Rettungsdienstes. Hierunter werden alle Maßnahmen verstanden, die vor Eintritt eines Ereignisses stattfinden, um der Entstehung von Notfällen vorzubeugen. Im Ergebnis soll das Risiko eines Notfallereignisses, welches zum Notruf 112 führt, verringert werden oder das Auftreten verzögert werden. Der vorbeugende Rettungsdienst soll auch dazu beitragen, das Outcome der medizinischen Versorgung von Patientinnen und Patienten zu verbessern. Weiterhin soll es ermöglicht werden, Hilfesuchende frühzeitig einer geeigneten Versorgungsform zuzuführen.

## Abstract (en)

In the Emergency Medical Service Acts of the Federal States, the statements in these Acts have so far essentially been limited to the implementation of measures to maintain the health of emergency patients and to transport them to a suitable hospital. Preventive fire protection, on the other hand, is regulated in the Fire Brigade Acts or by statutory ordinances. Increasing numbers of emergency service missions and a lack of facilities for alternative care justify the need for a preventive emergency service. This includes all measures that take place before an event occurs in order to prevent emergencies from occurring. As a result, the risk of an emergency event leading to the emergency call 112 should be reduced or delayed. The preventive rescue service should also help to improve the outcome of medical care for patients. Furthermore, it should be made possible to provide those seeking help with a suitable form of care at an early stage.

## Hintergrund

In den letzten Jahren sind die Anzahl der Notrufe als auch die Einsätze der Notfallrettung stetig angestiegen. Im Jahr 2021 sind beispielsweise in der Berliner Rettungsleitstelle insgesamt 1.095.932 Notrufe eingegangen, daraus resultierten 446.149 Einsätze der medizinischen Gefahrenabwehr [5]. Es wird wiederholt diskutiert, dass insbesondere niedrigprioritäre Hilfeersuchen einen wesentlichen Anteil der Einsätze des Rettungsdienstes ausmachen. In der Stadt Köln lag der Anteil an nicht akut lebensbedrohlichen Einsätzen in einem Zeitraum von einem Jahr konstant bei 60 % [45]. Hieraus folgt, dass auch das Rettungsdienstfachpersonal zu einem hohen Anteil mit niedrigprioritären Hilfeersuchen konfrontiert wird, teilweise gerät somit der Anteil derjenigen Einsätze, bei denen invasive bzw. heilkundliche Maßnahmen durchgeführt werden müssen, in den Hintergrund. In Zeiten von Fachkräftemangel ist ein Gefühl fehlender Sinnhaftigkeit von Einsätzen, einhergehend mit dem Gefühl höchster Einsatzbelastung, zunehmend problematisch [44]. Das Aufgabenspektrum des Rettungsdienstes wird sich voraussichtlich in den nächsten Jahren weiter verändern, und mangels Alternativen wird die Zuständigkeit für unkritische Patientinnen und Patienten mehr an Bedeutung gewinnen [56]. In dem Zusammenhang werden akut lebensbedrohliche Einsätze wohl auch zukünftig nur einen geringen Anteil an der Zahl der Gesamteinsätze ausmachen [68].

Die Notwendigkeit, das Rettungsdienstfachpersonal auch in sozialen Kompetenzen besser zu qualifizieren, wurde bereits durch den Gesetzgeber erkannt und in die Ausbildungs- und Prüfungsverordnung für Notfallsanitäterinnen und Notfallsanitäter (NotSan-APrV) aufgenommen. „*Kommunikation und Interaktion mit sowie Beratung von hilfesuchenden und hilfebedürftigen Menschen unter Berücksichtigung des jeweiligen Alters sowie soziologischer und psychologischer Aspekte*“ sind mit Einführung des Berufsbildes „Notfallsanitäter“ fest in dieser verankert [16].

Es fällt auf, dass präventive Ansätze in der Notfallrettung in Deutschland bislang weder gesetzlich verankert sind; weiterhin gelangen nur vereinzelt Pilotprojekte zur Umsetzung. Der vorliegende Beitrag zeigt Entwicklungen und Strategien hin zu präventiven Ansätzen im Rettungsdienst. Hierbei werden bereits etablierte Strukturen aus Nachbarländern sowie einzelne Projekte diskutiert, weiterhin wird dargestellt, wie diese in einem vorbeugenden Rettungsdienst gebündelt werden könnten.

## Präventive Ansätze im Rettungsdienst

Unter Prävention sind im Gesundheitswesen zielgerichtete Maßnahmen und Aktivitäten zu verstehen, die dazu dienen sollen, dass gesundheitliche Schädigungen oder Krankheiten vermieden werden. Weiterhin soll im Ergebnis das Auftreten von Erkrankungen verzögert oder weniger wahrscheinlich werden [57]. Auch im Bereich der Gefahrenabwehr spielt Prävention eine wesentliche Rolle. Neben der operativen Gefahrenabwehr gilt die vorbeugende Gefahrenabwehr als Teilbereich der Gefahrenabwehr, wobei es Zielsetzung ist, Gefahren und ihre Folgen zu minimieren [44]. Darunter fallen beispielsweise Aufgaben des vorbeugenden Brandschutzes, wobei die Zuständigkeit für den vorbeugenden Brandschutz in den Feuerwehrgesetzen bzw. durch Rechtsverordnungen geregelt ist. Im Gegensatz zu den Feuerwehrgesetzen beschränken sich die Ausführungen in den Rettungsdienstgesetzen der Länder bislang im Wesentlichen auf die Durchführung von Maßnahmen zum Erhalt der Gesundheit von Notfallpatientinnen und Notfallpatienten sowie auf die Beförderung in ein geeignetes Krankenhaus. Die Möglichkeit, Patientinnen und Patienten ambulant zu versorgen, ist nur in wenigen Rettungsdienstgesetzen gegeben. Darüber hinausgehende Regelungen, wonach der Rettungsdienst weitere Aufgaben der Gesundheitsvorsorge übernehmen kann, finden sich lediglich im Rettungsdienstgesetz des Saarlandes [51]. Insbesondere präventive Ansätze, die dazu dienen, dem Auftreten von Notfallereignissen oder medizinischen Hilfeersuchen an den Rettungsdienst entgegenzutreten, sind bislang kaum etabliert.

### Resilienzsteigerung der Bevölkerung

Der Bevölkerung scheint es zunehmend schwerzufallen, medizinische Hilfeersuchen adäquat einzuschätzen und daraus folgend das richtige Handeln zu schlussfolgern. In Bezug auf das Verhalten bei Notfällen scheint eine unzureichende Gesundheitskompetenz von Bedeutung zu sein, wobei sich Defizite insbesondere bei der Einschätzung der richtigen Versorgung bei nichtdringlichen Hilfeersuchen zeigen. Anhand einer Umfrage konnte gezeigt werden, dass gerade Patientinnen und Patienten mit inadäquater Gesundheitskompetenz eher eine zentrale Notaufnahme als den kassenärztlichen Bereitschaftsdienst aufsuchen würden, weiterhin ist auch die hausärztliche Anbindung bei diesen Patientinnen und Patienten schlechter [69].

Die bundeseinheitliche Rufnummer 116 117 des kassenärztlichen Bereitschaftsdienstes wurde im April 2012 deutschlandweit eingeführt. Mit der Einführung wurde bereits diskutiert, dass sich die hilfesuchenden Bürgerinnen und Bürger häufig schwertun einzuschätzen, ob es sich um eine lebensbedrohliche Situation handelt oder nicht [2]. Für das Land Berlin konnte im Rahmen einer Bevölkerungsumfrage festgestellt werden, dass nur 56 % der Befragten Alternativen zum Notruf 112 kennen und somit schlussgefolgert werden muss, dass gerade die fehlende Verfügbarkeit von Alternativen mitunter ursächlich für die ansteigende Frequenz von Notrufen ist [70].

Bereits seit vielen Jahren wird die Bevölkerung in Hinblick auf das Erkennen bestimmter bedrohlicher Erkrankungen, wie beispielsweise einem Schlaganfall, durch mediale Aufklärungskampagnen sensibilisiert. Es konnte gezeigt werden, dass derartige Kampagnen dazu führen, dass die Einsatzzahlen ansteigen [9, 53]. Demgegenüber stehen vermehrt Kampagnen, die dazu dienen sollen, die Bevölkerung im Hinblick auf einen verantwortungsvollen Umgang mit dem Notruf 112 zu sensibilisieren [4]. Weiterhin ist zu vermuten, dass ein veränderter Umgang mit dem Internet und sozialen Medien ebenfalls eine Rolle spielen, da auf diese Weise medizinische Laien bereits Verdachtsdiagnosen stellen und nach Lösungsansätzen suchen.

Im Ergebnis einer Notrufkampagne konnte die Frequenz von eingehenden Notrufen reduziert werden; weiterhin wurde die Anbindung an alternative Versorgungsformen erhöht [8]. Allerdings muss davon ausgegangen werden, dass derartige Kampagnen nur einen bestimmten Anteil der Bevölkerung erreichen. Hierbei ist auch zu berücksichtigen, dass nicht der Effekt erzielt werden darf, dass diejenigen, bei denen ohnehin schon die Schwelle, den Notruf zu wählen, erhöht zu sein scheint (z. B. ältere Menschen), gehemmt sind, dann bei einem lebensbedrohlichen Ereignis die 112 zu wählen [1].

Darüber hinaus wurden in den letzten Jahren vermehrt Bemühungen aufgenommen, neben der Aufklärung der Bevölkerung bzw. der Stärkung der Selbsthilfefähigkeit Erste-Hilfe-Kurse zu bewerben sowie Reanimationsunterricht in Schulen zu etablieren. In dem Zusammenhang ist erwähnenswert, dass zwar der verbindliche Reanimationsunterricht in Schulen ab der Klasse 7 im Umfang von 2 Doppelstunden/Jahr bereits im Jahr 2014 seitens des Schulausschusses der Kultusministerkonferenz befürwortet wurde und seitens des German Resuscitation Council ein Curriculum vorgelegt wurde [33], eine flächendeckende sowie verbindliche Umsetzung jedoch längst nicht erfolgt ist. Für eine effektive Umsetzung sind jedoch zwingend eine gesetzliche Verankerung sowie die Sicherstellung der Finanzierung notwendig [29]. Weiterhin sind adressatengerechte Bildungsangebote für die Bevölkerung, die dazu dienen, die notfallbezogene Handlungskompetenz zu stärken, bislang nicht etabliert. So beschränken sich Erste-Hilfe-Kurse im Wesentlichen auf medizinische Maßnahmen, und die Kompetenzentwicklung erfolgt ausschließlich im Rahmen klassischer Unterrichtsformate. Diese Form der Ausbildung reicht vermutlich nicht aus, um die Gesundheits- und Handlungskompetenz hinreichend zu stärken. Denkbar sind darüber hinaus insbesondere auch Kursformate, die die psychosoziale Notfallkompetenz stärken, weiterhin aber auch die Selbsthilfefähigkeit ganz allgemein mehr gewichten.

Auch Ersthelferalarmierungssysteme spielen eine immer wichtigere Rolle und tragen vermutlich zur Stärkung der individuellen Notfallkompetenz bei. Derartige Systeme sind bereits in vielen Kreisen und kreisfreien Städten etabliert worden, auch hier besteht jedoch das Erfordernis, die Finanzierung durch eine gesetzliche Grundlage zu regeln. In den Leitlinien zur Reanimation 2021 des European Resuscitation Council (ERC) wird im Kapitel „Lebensrettende Systeme“ explizit gefordert, dass Ersthelfer*innen, die sich in der Nähe eines vermuteten prähospitalen Herz-Kreislauf-Stillstandes aufhalten, über eine Smartphone-App alarmiert werden und somit derartige Technologien implementiert werden sollen, um die Zeit bis zur ersten Thoraxkompression und Defibrillation zu verkürzen. Es obliegt es den Kommunen und der Politik, die Umsetzung entsprechend zu fördern und voranzutreiben [38]. Damit einhergehend scheint auch die grundsätzliche Einbindung von Spontanhelfenden über derartige Technologien einen sinnvollen Ansatz darzustellen, der möglicherweise dazu dienen kann, die „community resilience“ nachhaltig zu stärken und tragfähige soziale Netzwerke zu bilden.

## Rolle der Leitstelle als zentrales Steuerungsorgan

Den Leitstellen des Rettungsdienstes kommt eine zentrale Rolle in der medizinischen Gefahrenabwehr zu. Neben der Notrufabfrage mit Anleitung zu Sofortmaßnahmen (z. B. Telefonreanimation) erfolgt dort die Disposition von Einsatzmitteln der Notfallrettung und teilweise auch von Einsatzmitteln des Krankentransportes. Zunehmend werden darüber hinaus die Leitstellen mit Hilfeersuchen konfrontiert, die zumindest teilweise nicht adäquat mit einem Einsatzmittel der Notfallrettung beantwortet werden können. Dementsprechend ist davon auszugehen, dass sich die Leitstellen hierzulande, in Analogie zu anderen europäischen Ländern, hin zu Beratungsinstanzen für sonstige medizinische und soziale Notsituationen entwickeln [3]. Auch wenn die Systeme im internationalen Vergleich sehr unterschiedlich sind, so ist die primäre telefonische Kontaktaufnahme mit der Leitstelle als zentrales Steuerungsorgan bereits in vielen europäischen Ländern fester Bestandteil der notfallmedizinischen Versorgung.

Bislang gibt es nur wenige Daten zur Frage, ob mittels der Anwendung einer standardisierten Notrufabfrage vorausgesagt werden kann, ob Hilfeersuchen mit niedriger Dringlichkeit dahingehend identifiziert werden können, ob sie eine prähospitale Behandlung durch den Rettungsdienst benötigen. Dennoch gibt es Hinweise, dass derartige Systeme zuverlässig sind [35]. Hieraus folgt, dass zur optimalen Ausnutzung von Ressourcen und um zukünftigen Herausforderungen gewachsen zu sein, die Notwendigkeit besteht, standardisierte Notrufabfrageprotokolle zu nutzen, die es ermöglichen, im Ergebnis der Notrufabfrage Dringlichkeiten zu klassifizieren.

Es bleibt aber oftmals – zumindest nach dem erstmaligen Notruf – die einzige Möglichkeit, ein Rettungsmittel zu entsenden, da gerade Patientinnen und Patienten mit unspezifischen Beschwerden ein erhöhtes Mortalitätsrisiko zu haben scheinen, sodass eine Versorgung in einer Notaufnahme resultieren muss [42].

## Zusammenarbeit mit anderen Partnern im Gesundheitswesen

Der Schnittstellenarbeit zu anderen Einrichtungen der Gesundheitsversorgung, auch außerhalb von Einsätzen, kommt eine zentrale Bedeutung zu. Die bundeseinheitliche Rufnummer der kassenärztlichen Vereinigung (KV) ist in der Bevölkerung nicht hinreichend bekannt, weiterhin gelingt es häufig nicht, die Dringlichkeit eines medizinischen Akutfalls richtig einzuschätzen und die passende Versorgungsentität für die medizinische Behandlung auszuwählen [49]. Die Problematik, dass trotz eines weit entwickelten Gesundheitssystems in Deutschland die Sektoren Rettungswesen und ambulante sowie stationäre Notfallversorgung nicht hinreichend miteinander vernetzt sind, wurde in den letzten Jahren seitens der verschiedenen Institutionen und Verantwortungsträger zunehmend erkannt und inzwischen auch auf der politischen Ebene aufgearbeitet. Hierbei besteht die Schwierigkeit mitunter darin, dass die Finanzierung der unterschiedlichen Leistungen völlig inhomogen erfolgt. Somit wird seitens des Bundesministeriums für Gesundheit ein neues System der integrierten Notfallversorgung gefordert, wobei hierzu im Jahr 2020 der Entwurf eines Gesetzes zur Reform der Notfallversorgung vorgelegt wurde [15]. Eine wesentliche Rolle spielt hierbei auch die Entwicklung hin zu einem gemeinsamen Notfallleitsystem, da vorgeschlagen wird, dass sowohl die Telefonnummer 116 117, als auch die 112 unmittelbar zueinander vernetzt sind. Auch wenn die KVen angehalten sind, eine telefonische Ersteinschätzung zu nutzen und darüber hinaus hierfür bereits eine strukturierte medizinische Ersteinschätzung für Deutschland entwickelt wurde, ist festzustellen, dass eine bundesweite Umsetzung noch nicht erfolgt ist [34]. Darüber hinaus existieren vielerorts noch keine funktionalen Schnittstellen zwischen den Einsatzleitsystemen der Rettungsleitstellen und der KV. Auch muss an dieser Stelle erwähnt werden, dass vermutlich auch lange Wartezeiten auf den kassenärztlichen Notdienst dazu beitragen, dass letztlich in vielen Fällen dennoch eine Alarmierung des Rettungsdienstes stattfindet.

Zuletzt zeigte sich eine veränderte Inanspruchnahme der ambulanten Notfallversorgung, wobei Rückgänge in den letzten Jahren im Zusammenhang mit einer 24/7-Erreichbarkeit und der Einführung der strukturierten medizinischen Ersteinschätzung im Jahr 2019 diskutiert werden [48].

## Hilfeersuchen durch Pflegeeinrichtungen

Es wird immer wieder diskutiert, dass Krankenhauszuweisungen aufgrund von Notrufen durch Pflegeeinrichtungen in vielen Fällen vermeidbar sind [10]. Ursächlich konnten als einweisungsbegünstigende Umstände einrichtungsbezogene (Personalfluktuation, Unsicherheiten), aber auch systembedingte Umstände sowie die mangelnde Erreichbarkeit von Ärztinnen und Ärzten identifiziert werden [10]. Hierbei kommt der Verbesserung der Kommunikation und dem gegenseitigen Verständnis zwischen den verschiedenen Professionen eine entscheidende Bedeutung zu, da insbesondere aus der Perspektive der „Pflegekräfte“ keineswegs vorschnell eine Alarmierung des Rettungsdienstes erfolgt [61]. Zwar wurden beispielsweise „Katheterprobleme“ als potenziell vermeidbar eingestuft, vielerorts scheinen jedoch schlichtweg heiminterne Handlungsanweisungen und die fehlende Verfügbarkeit von fachärztlichem Personal ursächlich zu sein [61]. Weiterhin wurde bereits identifiziert, dass unterschiedliche Wahrnehmungen von Notfallsituationen ohnehin zu Missverständnissen in der interprofessionellen Zusammenarbeit führen können, wobei auch Unsicherheiten und Ängste bei der Bewertung eine Rolle spielen [62].

Hieraus folgt also, dass es zielführend sein kann, abgestimmte Handlungsanweisungen zu erstellen, indem die Schnittstellenarbeit intensiviert und optimiert wird.

Als weiteres Beispiel sind *Frequent Fallers* zu nennen, die durch Stürze in Pflegheimen oder anderen organisierten Wohnformen wiederholt Einsätze des Rettungsdienstes verursachen, ohne dass die Notwendigkeit eines Transportes in ein Krankenhaus besteht [19]. Auch hier kann durch eine frühzeitige Kommunikation die Schnittstellenarbeit verbessert werden. In dem Zusammenhang konnten beispielsweise Rettungskräfte als geeignet identifiziert werden, um die Anbindung an entsprechende Sturzpräventionsprogramme sicherzustellen [27]. Insbesondere die Problematik, dass über das System der Notfallrettung bei *Frequent Fallers* in keiner Weise eine Nachsorge sichergestellt ist, um zukünftige Stürze zu verhindern, führt offensichtlich zu wiederholten Einsätzen [63].

## Innovative Einsatzmittel

Es gibt Hinweise darauf, dass ambulante Kontakte mit dem Rettungsdienst genauso wie ambulante Kontakte in Notaufnahmen in den letzten Jahren kontinuierlich angestiegen sind [36]. Gerade Einsätze, die als Behandlung vor Ort abgeschlossen wurden, haben zugenommen [64]. Aus einer Stellungnahme des Bundesverbandes der Ärztlichen Leitungen Rettungsdienst in Deutschland aus dem Jahr 2015 geht hervor, dass der Bundesverband Rettungsdienstfachpersonal abrät, Patientinnen und Patienten mit banalen Erkrankungen oder Verletzungen zu Hause zu lassen. Hintergrund sind demnach Limitationen in der prähospitalen Diagnostik und die damit einhergehenden erhöhten Anforderungen an die Sorgfaltspflicht, insbesondere unter Würdigung drohender strafrechtlicher Konsequenzen [13]. In einer neueren Checkliste aus dem Jahr 2017 ist die Einholung einer ärztlichen Entscheidung durch das Rettungsdienstfachpersonal empfohlen [14].

Ein grundlegendes Hindernis besteht darin, dass der Rettungsdienst aktuell aufgrund der Verankerung im Sozialgesetzbuch als „Transportleistung“ klassifiziert ist und dementsprechend Einsätze grundsätzlich nur dann seitens der Krankenkassen erstattet werden, wenn auch ein Transport mit entsprechender Krankenhausbehandlung erfolgt ist. Auch wenn das „Notfallreformgesetz“ vorsieht, dass der Rettungsdienst eigener Leistungsbereich der gesetzlichen Krankenversicherungen wird, sind auch die Regelungen in den Landesrettungsdienstgesetzen sehr unterschiedlich. Aus den wenigsten Landesrettungsdienstgesetzen ist in Bezug auf die Notfallrettung die Möglichkeit einer ambulanten Behandlung aus der Aufgabenbeschreibung überhaupt herzuleiten.

Allerdings ist das alleinige „Nichttransportieren“ von Hilfesuchenden dahingehend nicht zielführend, dass das Problem damit nicht oder nur kurzfristig gelöst ist. Somit sucht mehr als die Hälfte derjenigen Patientinnen und Patienten, die durch den Rettungsdienst zu Hause gelassen werden, gerade bei „unspezifischen Symptomen“, innerhalb von 72 h erneut eine Gesundheitseinrichtung auf [31].

Bezugnehmend auf das Rettungsdienstfachpersonal gibt es bislang kaum Untersuchungen dahingehend, ob es gelingt einzuschätzen, wann Patientinnen und Patienten in ein Krankenhaus transportiert werden müssen und wann nicht [32]. In einem Paramedic-System konnte festgestellt werden, dass es zu signifikanten Unter- und Übertriagierungen im Vergleich zu Ärztinnen und Ärzten kommt [52]. Weiterhin gab es gerade in Paramedic-Systemen wiederholt Studienergebnisse, die darauf hinweisen, dass eine derartige Einschätzung Paramedics nur unzureichend gelingt [12, 65]. Bei einem Teil der Patientinnen und Patienten, die nicht transportiert werden, erfolgt innerhalb von 2 Tagen eine erneute Vorstellung [25]. Allerdings ist davon auszugehen, dass schwerwiegende Ereignisse zum Nachteil von Patientinnen und Patienten eher die Ausnahme darstellen [18]. Weiterhin konnte eine erhöhte Sterblichkeit im Zusammenhang mit ambulanter Versorgung vor Ort bislang nicht nachgewiesen werden [36, 37].

Problematisch erscheint dennoch die adäquate prähospitale Einschätzung weniger dringlich kategorisierter Patientinnen und Patienten durch Rettungsdienstfachpersonal. Eine Untersuchung aus Deutschland ergab, dass bei über 70 % der als „weniger dringlich“ kategorisierten Patienten in der Notaufnahme eine Heraufstufung im Manchester Triage System erfolgen musste [46]. Somit ist es erforderlich, dass auch dahingehend Aus- und Fortbildung erfolgt und insbesondere Protokolle und Checklisten vorgehalten werden, um die Patientensicherheit bestmöglich zu gewährleisten.

Die zuvor genannten Entwicklungen führen zwangsläufig dazu, dass es nicht zielführend ist, alle Patientinnen und Patienten in ein Krankenhaus zu transportieren. Allerdings existieren in einem großstädtischen Rettungsdienst zurzeit hohe technische, organisatorische und rechtliche Hürden, Patienten in eine krankenhausalternative Versorgungseinrichtung durch den Rettungsdienst zuzuweisen [54]. Standardarbeitsanweisungen (SAA) bzw. Standard Operating Procedures (SOP) hingegen, die die Möglichkeit eröffnen, Patientinnen und Patienten ambulant zu versorgen, erscheinen umsetzbar und notwendig. Hierbei müssen die Optimierung von Notrufabfrageprotokollen und die Entwicklung der Leitstellentechnologie sowie die Anbindung von Telemedizin eine Rolle spielen [22, 23]. Mittels einer klareren Indikationsstellung und des Einsatzes von entsprechenden Technologien kann die Verfügbarkeit von Notärzten optimiert werden und der Anteil von physischen Arztkontakten reduziert werden [28].

Sinnvoll ist die unmittelbare Integration von Lotsen hin zu alternativen Versorgungswegen in den Rettungsdienst, beispielsweise durch „community paramedics“ (Gemeindenotfallsanitäter oder NotSan-Erkunder). Bezugnehmend auf alternative Versorgungswege gibt es Hinweise auf eine verbesserte Effizienz im Zusammenhang mit einer derartigen patientenzentrierten Versorgung [6, 20, 55].

Um der Problematik von teilweise undifferenzierten Krankenhauszuweisungen und der fehlenden Versorgungsmöglichkeit vor Ort entgegenzutreten, wurde im Oldenburger Land das Projekt „Gemeindenotfallsanitäter“ initiiert. Hierbei werden speziell geschulte Notfallsanitäter zu ausgewählten Hilfeersuchen disponiert, wobei diesen vor Ort einerseits die Aufgabe der Sichtung, aber auch eine Zuweisungs- und Behandlungskompetenz zukommt [30]. In einer ersten Beobachtungsstudie konnte gezeigt werden, dass in 59 % der Fälle keine dringliche Versorgung vorgelegen hat. In 59 % der Fälle war ein nachfolgender Transport in eine Klinik nicht notwendig [60]. Auch in Berlin wurden im Rahmen der COVID-19-Pandemie NotSan-Erkunder als innovatives Einsatzmittel eingeführt [11]. Bei *Frequent Usern, *also Menschen die wiederholt den Rettungsdienst in Anspruch nehmen, konnte ebenfalls ein Nutzen derartiger Einsatzmittel gezeigt werden [7]. In Niederösterreich wurden als Pilotprojekt mit Datum vom 28.11.2019 „acute community nurses“ zur Unterstützung des Rettungsdienstes etabliert. Hierbei geht es in erster Linie darum, adäquat auf den steigenden psychosozialen und pflegerischen Bedarf in Akutsituationen reagieren zu können und somit Transporte in Kliniken zu vermeiden.

Weiterhin sind Ansätze denkbar, ambulante hausärztliche Versorgung an den Rettungsdienst anzubinden, um auf diese Weise ausgewählte Patientinnen und Patienten in der Häuslichkeit aufzusuchen [41].

Im europäischen Ausland gibt es bereits vielerorts Projekte, die vergleichbare Ziele haben. So wurde in Kopenhagen, Dänemark, eine sog. Sozialambulanz („Sociolance“) etabliert [43]. Hierbei konnte gezeigt werden, dass diese insbesondere von Menschen mit Suchterkrankung oder psychosozialem Problem in Anspruch genommen wird, wobei neben Transporten ins Krankenhäuser auch Transporte in Notunterkünfte durchgeführt werden. Im Ergebnis eines Pilotprojektes konnten 9 von 10 Nutzerinnen oder Nutzern an alternative Versorgungsformen angebunden werden, weiterhin nahmen 3 von 4 Nutzerinnen und Nutzern das Angebot auch an [43]. Bei den Nutzerinnen und Nutzern handelt es sich um eine sehr heterogene Gruppe mit komplexen sozialen und gesundheitlichen Problemen. Es wurde aber auch festgestellt, dass das Fehlen von verfügbaren Behandlungsangeboten limitierend ist. Die Herausforderung besteht somit insbesondere darin, im Hintergrund ein adäquates Netzwerk aufzubauen, um überhaupt entsprechende Hilfestellungen leisten zu können [66].

Darüber hinaus werden an den Rettungsdienst vermehrt sozialpsychiatrische Fragestellungen adressiert. Schnittstellen zu sozialpsychiatrischen Diensten existieren bisher nicht, weiterhin ist eine niedrigschwellige Verfügbarkeit von sozialpsychiatrischem Fachpersonal regelhaft nicht gegeben. In vielen Fällen kann der Rettungsdienst derartige Fragestellungen auf der Ebene der Leitstelle nur mittels Entsendung eines Einsatzmittels und auf der Ebene der operativen Einsatzbearbeitung nur mittels Transport in ein Krankenhaus begegnen. Auch aufgrund der fehlenden Fachexpertise ist der Transport in eine Klinik oft die einzige Lösung. Hierbei wird oft lediglich in das nächstgelegene somatische Krankenhaus transportiert, sodass, sofern sich nach entsprechender Diagnostik und Untersuchung keine Indikation zur stationären Aufnahme ergeben hat, die Entlassung in die Häuslichkeit erfolgt, ohne dass eine Anbindung an eine geeignete ambulante Versorgung erfolgt ist.

Beim London Ambulance Service werden seit dem Jahr 2015 „mental health nurses“ in der Leitstelle eingesetzt. Hier kann somit eine Risikobewertung nach erfolgter standardisierter Notrufabfrage erfolgen, weiterhin aber auch die Unterstützung bei der Anbindung an eine geeignete Hilfseinrichtung [24, 47]. Fachkundiges Personal steht auf diese Weise zum einen als Unterstützung für die Disponenten in der Leitstelle, aber auch für Besatzungen von Rettungswagen zur telefonischen Unterstützung zur Verfügung. In ersten Untersuchungen konnte eine Verringerung der Beschickung mit Rettungswagen nach Einführung des Pilotprojektes festgestellt werden [40].

Auch „mental health joint response cars“, die neben einem Paramedic mit einer Mental health nurse besetzt sind, befinden sich seit dem Jahr 2018 in London im Einsatz. Die Idee, sog. multiprofessionelle Kriseninterventionsteams als Reaktion auf Notrufe von Menschen, die sich in einem psychischen Ausnahmezustand befinden, an den Rettungsdienst anzubinden, wurde im Koalitionsvertrag für das Land Berlin für die aktuelle Legislaturperiode festgehalten [59].

## Case-Management

Im Gesundheitswesen in Deutschland haben sich im Entlassmanagement im stationären Bereich in den letzten Jahren vielerorts sog. Case-Manager als fester Bestandteil der Gesundheitsversorgung etabliert. Als Case-Manager wird *„eine Fachkraft bezeichnet, welche die Schnittstelle zwischen Ärzten, Pflegefachkräften und Therapeuten darstellt und deren Dienstleistungen für die Patienten organisiert und koordiniert“ *[58]. Diesem kommt somit eine wesentliche Aufgabe bei der Bedienung von Schnittstellen zwischen den verschiedenen Leistungssektoren und insbesondere der Integration in alle verfügbaren Behandlungseinrichtungen aus dem ambulanten und stationären Sektor zu.

Oft führen Defizite im Entlassmanagement binnen kürzester Zeit zum Wiedereintritt des Hilfeersuchens, da der Patientin/dem Patienten trotz Vorstellung im Krankenhaus nicht hinreichend geholfen wurde. Im Ergebnis erfolgt hieraus auch eine erhöhte Belastung von Notaufnahmen [26].

**Figure 1:**
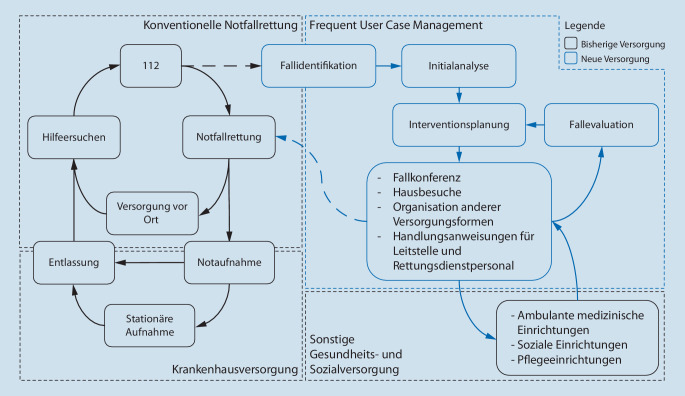

In den meisten Fällen wird die Funktion eines Case-Managers durch erfahrene Pflegekräfte oder Sozialarbeiter wahrgenommen. Hierbei spielt die interprofessionelle Abstimmung zwischen den verschiedenen Berufsgruppen eine entscheidende Rolle, weiterhin ist die frühzeitige Kommunikation sowohl mit den Patientinnen und Patienten als auch mit den Weiterbehandelnden entscheidend. Auch in der Versorgung von *Frequent Usern* ist in vielen Ländern ein Case-Management bereits fester Bestandteil der Primärversorgung. Weiterhin gibt es eine Reihe von Untersuchungen, die gezeigt haben, dass Case-Management sowohl die Einsatzzahlen im Zusammenhang mit *Frequent Usern* reduzieren kann, aber auch die Lebensqualität bei *Frequent Usern* erhöhen kann. Eine Möglichkeit der Anbindung eines Case-Managements an den Rettungsdienst zeigt Abb. 1. Multiprofessionelle Teams aus Ärzten, Sozialarbeitern, Pflegekräften und Rettungsdienstfachpersonal können helfen, einen integrativen, ganzheitlichen Ansatz darzustellen und die Versorgungsqualität durch flexible Einsatzmöglichkeiten zu verbessern. Hierbei kann das zu etablierende Case-Management mit regelmäßig stattfindenden Fallkonferenzen in der Aufbauorganisation eines vorbeugenden Rettungsdienstes auch die zentrale Schlüsselrolle einnehmen (Tab. 1).

**Table 1:** 

Funktion	Qualifikation	Aufgabengebiet	Operativer Einsatz
Case-Management, Arzt	Ärztin/Arzt	Inhaltliche Verantwortung, Case-Management, Schnittstellenarbeit	–
Case-Manager, Sozialarbeiter	Sozialarbeiterin/Sozialarbeiter	Sachbearbeitung, Case-Management, Schnittstellenarbeit	Sozialambulanz
Case Management Nurse	Gesundheits- und Krankenpflege	Sachbearbeitung, Case-Management, Schnittstellenarbeit	Acute Community Nurse
Case-Management, NotSan	Notfallsanitäterin/Notfallsanitäter	Sachbearbeitung, Case-Management, Schnittstellenarbeit	Community Paramedic
Case-Management, Schnittstellenarbeit	Bachelor‑/Masterabsolventen Pflege/Gesundheitswissenschaften/Public Health	Sachbearbeitung, Case-Management, Schnittstellenarbeit	–

## Diskussion und Ausblick

Durch die Etablierung eines vorbeugenden Rettungsdienstes in der Organisationsstruktur soll die Schnittstellenarbeit zu anderen Versorgungsdiensten im Gesundheitswesen (Sozialdienst, Gesundheitsamt, Notaufnahmen) optimiert werden, weiterhin sollen auch Projekte zur Prävention sowie Pilotprojekte zu neuen Versorgungsformen in dem Bereich initiiert werden. Damit einhergehend ist es denkbar, hier die zuvor dargestellten Maßnahmen zu bündeln, die zur Stärkung der Gesundheitskompetenz der Bevölkerung beitragen sollen. Ein gesetzlicher Auftrag in den Landesrettungsdienstgesetzen hierfür ist erforderlich, um auf der Basis auch eine Finanzierung sicherzustellen und die Patientensicherheit zu gewährleisten. Auf dieser Basis wäre auch die Einbindung von Aufsichtsbehörden zu diskutieren, sofern einzelne Einrichtungen, z. B. Pflegeheime, ihrem Auftrag nicht nachkommen und es dort wiederholt zu vermeidbaren Rettungsdiensteinsätzen kommt.

Einzelne Rettungsdienstgesetze sehen bereits vor, dass geeignete Qualitätsmanagementstrukturen geschaffen werden, um anhand einer differenzierten Datenerfassung und -auswertung unter Mitwirkung aller Beteiligten eine regelmäßige Analyse der Struktur‑, Prozess- und Ergebnisqualität durchführen zu können [50]. Weiterhin existieren beispielsweise in den Bundesländern Baden-Württemberg und Bayern Strukturen, die jährlich umfassende Qualitätsberichte – auch öffentlich – zur Verfügung stellen [39, 67] Hierbei liegt der aktuelle Fokus jedoch im Wesentlichen auf der Darstellung von Qualitätsindikatoren und Kennzahlen, die sich auf planerische Hilfsfristen oder Tracer-Diagnosen beziehen. Eine systematische Berichterstattung mit Betrachtung von Fehleinsätzen, Einsätzen mit ambulanter Versorgung oder Abgaben an alternative Versorgungsformen erfolgt bislang nicht. Ein derartiges Berichtswesen sollte zwingend unter Würdigung oben genannter Punkte gesetzlich verankert werden.

Die Berliner Feuerwehr hat im Dezember 2020 mit einer Umstrukturierung begonnen, die auch eine Neuorganisation der Abteilung Rettungsdienst, die von der Ärztlichen Leitung Rettungsdienst verantwortet wird, beinhaltet. In dem Zusammenhang wurde die Entwicklung eines „vorbeugenden Rettungsdienstes“ erstmalig im Rahmen der Projektphase aufgegriffen und letztlich auch als eigenes Referat in der Abteilung Rettungsdienst fest verankert. Zwischenzeitlich wurde die Zielsetzung, Regelungen für einen vorbeugenden Rettungsdienst zu schaffen, auch in die Koalitionsvereinbarung von CDU und Grünen für das Bundesland Nordrhein-Westfalen aufgenommen [17].

Als eine der wesentlichen Aufgaben des vorbeugenden Rettungsdienstes in Berlin wurde zunächst der Fokus auf die „*Prävention durch Aufklärung und Stärkung der Bevölkerung und von Institutionen der Gesundheits- und Notfallversorgung“ *gelegt [21].

Eine umfassende Definition des vorbeugenden Rettungsdienstes zeigt die Infobox.

**Figure 2:**
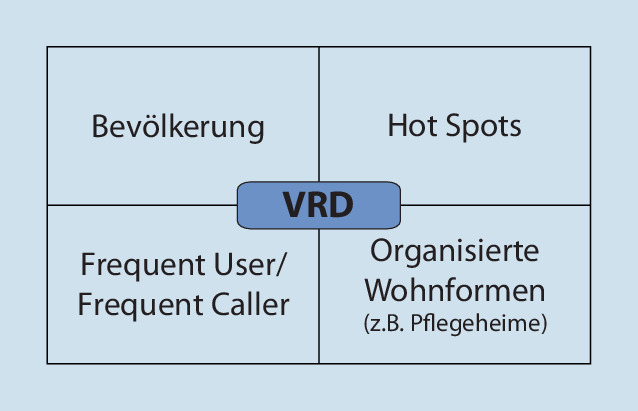

**Figure 3:**
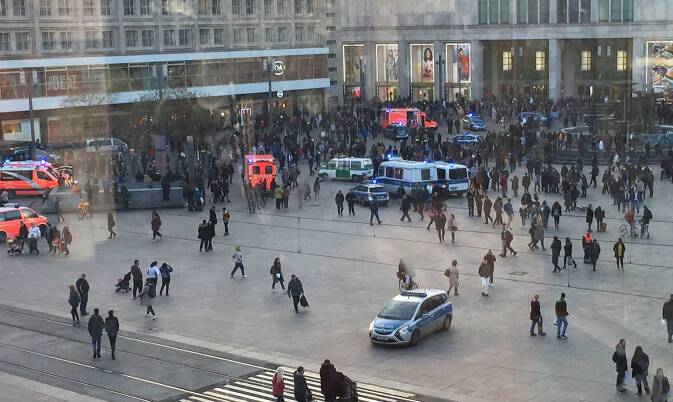

Diejenigen Themenfelder, die mit Einführung des vorbeugenden Rettungsdienstes in der Berliner Notfallrettung initial identifiziert worden sind zeigt Abb. 2. Hierbei wurde u. a. zugrunde gelegt, dass es gerade im urbanen Rettungsdienst an bestimmten *Hot Spots* im Zusammenhang mit Versammlungen oder an besonderen Tagen immer wieder zu Einsätzen der Notfallrettung kommt. Hierbei kann ein *Hot Spot* zum einen ein ständig touristisch aufgesuchter „Sightseeing-Schwerpunkt“ oder ein Bahnhof sein, andererseits aber auch temporär eine Partymeile an einem Wochenende oder ein bestimmter Bereich aufgrund einer besonderen Sportveranstaltung wie einem Marathon (Abb. 3). Hier spielt auch die Zusammenarbeit mit den Ordnungsbehörden bei der Bewertung von Einsatzplänen bzw. Sicherheitsgutachten von Veranstaltungen mit erhöhtem Gefährdungspotenzial eine Rolle, darüber hinaus können auch straßenbauliche Maßnahmen unmittelbar Einfluss auf Anfahrten und Rettungswege haben, die in dem Zusammenhang berücksichtigt werden müssen.

Neben Schwerpunkten im Zusammenhang mit obdachlosen/wohnungslosen Menschen liegt der Fokus auf *Frequent Usern*, denen oft durch den alleinigen Transport in ein Krankenhaus nicht hinreichend geholfen ist.

Darüber hinaus erscheint es notwendig, auch die Aus- und Fortbildung des Rettungsdienstfachpersonals, insbesondere der Notfallsanitäter, weiter an die systemischen Veränderungen anzupassen und damit einhergehend auch Veränderungen im beruflichen Selbstverständnis herbeizuführen. Letztlich ist festzustellen, dass sich trotz der Anpassungen der NotSan-APrV, das Rettungsdienstfachpersonal für eine Vielzahl von Einsätzen der Notfallrettung schlichtweg nicht zuständig fühlt und darüber hinaus insbesondere seitens der Berufsverbände dahingehend auch kaum Bemühungen erkennbar sind, eine Weiterentwicklung des Berufsbildes über das notfallmedizinische Sachverständnis und die Behandlung von akuten Notfällen hinaus herbeizuführen.

### Infobox Definition „vorbeugender Rettungsdienst“

Unter vorbeugendem Rettungsdienst werden alle Maßnahmen verstanden, die vor Eintritt eines Ereignisses stattfinden, um der Entstehung von Notfällen vorzubeugen. Im Ergebnis soll das Risiko eines Notfallereignisses, welches zum Notruf 112 führt, verringert werden oder das Auftreten verzögert werden. Der vorbeugende Rettungsdienst soll auch dazu beitragen, das Outcome der medizinischen Versorgung von Patientinnen und Patienten zu verbessern. Weiterhin soll es ermöglicht werden, Hilfesuchende frühzeitig einer geeigneten Versorgungsform zuzuführen.

## Fazit für die Praxis

- In Analogie zum vorbeugenden Brandschutz sollte bundesweit auch im Rettungsdienst ein präventiver Ansatz verfolgt werden, um vermeidbare Notrufe und Einsätze zu reduzieren und Patientinnen und Patienten frühzeitig einer geeigneten Versorgungsform zuzuführen.
- Es sollten Strukturen aufgebaut werden, die es ermöglichen, durch Aus- und Fortbildung, Aufklärung, Information und Vernetzung das Auftreten von Ereignissen, die zum Notruf 112 führen, zu reduzieren sowie die Gesundheits- und Handlungskompetenz der Bevölkerung zu stärken.
- Zwingend erforderlich ist die Verankerung in den Landesrettungsdienstgesetzen, um Regelungen zur Finanzierung zu schaffen und eine einheitliche Versorgungsqualität sicherzustellen.
